# Development of a novel encystment medium: Enhancing diagnostic potential of *Acanthamoeba* spp.

**DOI:** 10.14202/vetworld.2025.110-121

**Published:** 2025-01-22

**Authors:** Julalak Chuprom, Suthinee Sangkanu, Watcharapong Mitsuwan, Rachasak Boonhok, Alok K. Paul, Sonia M. Rodrigues Oliveira, Maria L. Pereira, Tajudeen O. Jimoh, Mohammed Rahmatullah, Polrat Wilairatana, Christophe Wiart, Ajoy K. Verma, Veeranoot Nissapatorn

**Affiliations:** 1 Department of General Education, School of Languages and General Education, Walailak University, 222 Thaiburi, Thasala, Nakhon Si Thammarat, Thailand, 80160; 2 School of Allied Health Sciences, Southeast Asia Water Team (SEA Water Team) and World Union for Herbal Drug Discovery (WUHeDD), Walailak University, Nakhon Si Thammarat, Thailand; 3 Akkhraratchumari Veterinary College, Walailak University, Nakhon Si Thammarat, Thailand; 4 Department of Medical Technology, School of Allied Health Sciences, Research Excellence Center for Innovation and Health Products, Walailak University, Nakhon Si Thammarat, Thailand; 5 School of Pharmacy and Pharmacology, University of Tasmania, Hobart, TAS, Australia; 6 Faculty of Dental Medicine, the Catholic University of Portugal; 7 Department of Medical Sciences, CICECO-Aveiro Institute of Materials, University of Aveiro, Aveiro, Portugal; 8 Department of Microbiology, Icahn School of Medicine at Mount Sinai, New York, USA; 9 Department of Biotechnology and Genetic Engineering, University of Development Alternative, Lalmatia, Dhaka, Bangladesh; 10 Department of Clinical Tropical Medicine, Faculty of Tropical Medicine, Mahidol University, Bangkok, Thailand; 11 The Institute for Tropical Biology and Conservation, Kota Kinabalu, Sabah, Malaysia; 12 Department of Microbiology, National Institute of Tuberculosis and Respiratory Diseases, New Delhi, India; 13 Futuristic Science Research Center, School of Science, Walailak University, Nakhon Si Thammarat, Thailand

**Keywords:** *Acanthamoeba*, diagnostic tools, encystation, medium optimization, response surface methodology

## Abstract

**Background and Aim:**

*Acanthamoeba* spp. are pathogenic microorganisms linked to severe infections in humans and animals, requiring a deeper understanding of their encystation process for effective diagnostics and research. This study focused on developing a novel encystment medium to induce synchronized encystation of *Acanthamoeba* spp. efficiently and rapidly.

**Materials and Methods:**

The study employed response surface methodology with a central composite design to optimize the encystment medium formulation. The key components included Tris-HCl, NaCl, glucose, and MgCl_2_. The optimized liquid medium was spray-dried to produce a dehydrated powder for practical application. The encystation efficiency of different *Acanthamoeba* strains was assessed using hemocytometry and fluorescence microscopy.

**Results:**

The optimized medium, comprising 3.152 g/L Tris-HCl, 5.55 g/L NaCl, 8% (w/v) glucose, and 5.0 mM MgCl_2_ at pH 9.0, demonstrated exceptional encystation efficiency with rates ranging from 99% to 100%. A spray-dried powdered version of this medium was equally effective, achieving a 98.77% encystation rate for *A. castellanii* American Type Culture Collection 50739 in glucose-free conditions. Notably, optimal glucose concentrations varied among *Acanthamoeba* strains, with certain strains reaching maximum encystation at 6–8% glucose.

**Conclusion:**

This study successfully developed an innovative encystment medium that promotes rapid and efficient cyst production in *Acanthamoeba* spp. The medium enhances laboratory research and diagnostic capabilities, paving the way for future advancements in understanding and managing *Acanthamoeba* infections.

## INTRODUCTION

*Acanthamoeba* spp. are free-living amoeba commonly found in various environmental resources, such as soil and water [1, 2]. They can cause rare and fatal brain infections of the central nervous system, also known as granulomatous amoebic encephalitis (GAE). The mortality rate associated with *Acanthamoeba*-induced GAE is extremely high, exceeding 90%, and it predominantly affects individuals with metabolic, physiological, and immunological disorders [3]. *Acanthamoeba* is responsible for corneal infections and can lead to blindness, a condition referred to as *Acanthamoeba* keratitis (AK), often associated with poor contact lens (CL) hygiene practices among CL users. Furthermore, strong evidence exists that AK exists in animals [4, 5]. *Acanthamoeba* transitions between two stages in its life cycle, from active trophozoites to dormant cysts [6]. During the trophozoite stage, the parasite, ranging in size from 15 to 50 μm and assuming irregular, oval, round, or pear-like forms, prominently displays acanthopodia-spine-like protrusions on its surface – that actively assist its attachment to CLs [2]. Encystment is a common stress response observed in many protists, including *Acanthamoeba*, to protect against unfavorable conditions, such as malnutrition, exposure to biocides, extreme temperatures, pH variations, chlorine presence, and anti-*Acanthamoeba* agents [7, 8]. *Acanthamoeba* undergoes morphological and biochemical changes during encystation, leading to reduced metabolic activity [9]. *Acanthamoeba* encystation comprises three phases: induction, wall synthesis, and dormancy [10, 11]. With the increasing incidence of *Acanthamoeba* infections in recent years, establishing an appropriate protocol for this unique organism is challenging due to its dual life stage, effective cultivation methods, encystment techniques, and other factors that can affect test outcomes.

The process of inducing encystment in *Acanthamoeba* can be experimentally achieved through various laboratory protocols, including the use of encystment-promoting media like non-nutrient agar (NNA), and maintaining cultures at 28°C for up to 14 days [12, 13]. Neff’s encystment medium (NEM) has also been used to induce encystment in *Acanthamoeba*, resulting in the formation of mature cysts after 1 week of cultivation at 25°C [14]. However, for research on *Acanthamoeba* cysts, it is essential to obtain synchronized cultures in which the majority of trophozoites transform into cysts [13]. Prolonged axenic cultivation of *Acanthamoeba* can lead to various alterations affecting amoebic virulence [15], cellular enzyme activity changes, drug sensitivity [16, 17], loss of encystment ability [18], and reduced temperature tolerance [19]. It is plausible that long-term culture impacts *Acanthamoeba* encystment capacity. Therefore, the development of a medium that facilitates rapid encystment of *Acanthamoeba* is of paramount importance to ensure reliable test results. Medium optimization using conventional methods, specifically one-factor-at-a-time (OFAT) techniques, is often time-consuming and fails to consider the interactive effects between factors. This method requires a significant number of experimental datasets to achieve satisfactory results [20]. However, these limitations can be overcome by utilizing response surface methodology (RSM). RSM is beneficial for identifying key factors, minimizing the number of required experiments, and determining optimal conditions for production [21, 22]. RSM, which involves mathematical and statistical techniques, has been successful in optimizing various processes, including enzyme production, microbial growth rate enhancement, and bioethanol production [23, 24]. To the best of our knowledge, no studies have been published on the development of dehydrated encystment media.

This study aimed to achieve the following objectives: (i) develop a novel encystment medium and optimal conditions for the highest production of mature amoeba cysts in the minimum required time using statistical tools, (ii) produce dehydrated medium powder from the novel liquid encystment medium using a spray dryer, and (iii) evaluate the effectiveness of this dehydrated medium powder in inducing encystation in *Acanthamoeba* spp. The results of this study have significant implications for the commercial advancement and application of the medium, especially for the rapid production of *Acanthamoeba* cysts.

## MATERIALS AND METHODS

### Ethical approval

The study was approved by the Committee of the Biosafety Guidelines for Scientific Research of Walailak University, Nakhon Si Thammarat, Thailand (Ref. No. WU-IBC-66-020).

### Study period and location

The study was conducted from October 2022 to October 2023 at Tropical Medicine Laboratory, Research Institute for Health Sciences (RIHS), and Center for Scientific and Technological Equipment, Walailak University, Nakhon Si Thammarat, Thailand.

### Chemicals

Proteose peptone, page’s saline, and yeast extract were purchased from HiMedia Laboratories (Mumbai, India). Calcofluor white (CFW) stains, sodium citrate dihydrate (C_6_H_5_Na_3_O_7_.2H_2_O), dibasic sodium phosphate heptahydrate (Na_2_HPO_4_.7H_2_O), sodium chloride (NaCl), calcium chloride (CaCl_2_), and glucose were obtained from Sigma Chemical Co. (St. Louis, MO, USA). Potassium dihydrogen phosphate (KH_2_PO_4_) and magnesium sulfate heptahydrate (MgSO_4_.7H_2_O) were purchased from Labscan (Bangkok, Thailand). Trypan blue (0.4%) was obtained from Gibco BRL (Grand Island, NY, USA). All chemicals and medium components used were of analytical grade.

### *Acanthamoeba* strains and growth conditions

*Acanthamoeba triangularis* WU19001 (MW647650) genotype T4 was isolated from the recreational reservoir of Walailak University by Mitsuwan *et al*. [25]. The isolates obtained from the American Type Culture Collection (ATCC) for use in these studies included *Acanthamoeba polyphaga* ATCC30461, *Acanthamoeba castellanii* ATCC50739, and *A. castellanii* ATCC30010. Each of the isolates used in this study was seeded in T-25 tissue culture flasks (SPL Life Science Co., Ltd., Gyeonggi-do, Korea) with 15 mL of peptone–yeast–glucose extract (PYG) medium [26] containing (g/L) proteose-peptone 20, yeast extract 2.0, and glucose 18, C_6_H_5_Na_3_O_7_.2H_2_O 1.0, MgSO_4_.7H_2_O 0.98, Na_2_HPO_4_.7H_2_O 0.355, KH_2_PO_4_ 0.34, and ammonium iron (II) sulfate hexahydrate [Fe(NH_4_)_2_(SO_4_)_2_.6H_2_O] 0.02 in 1000 mL of distilled water pH 7.0. After incubation in the dark at 28°C for 24 h, *Acanthamoeba* trophozoites were observed.

*Acanthamoeba* cysts were prepared by first growing the trophozoites in PYG medium for 24 h, followed by centrifugation at 1520× *g* for 5 min (Sorvall ST 40R centrifuge, Thermo Scientific, CA, USA). The resulting pellet was washed twice with an encystment medium. The trophozoite concentration was adjusted to 3 × 10^5^ trophozoites/mL and transferred to T-25 tissue culture flasks containing 15 mL of encystment medium. Encystation was induced by incubating in the dark at 28°C.

### Evaluation of different encystment media for inducing encystation using the OFAT approach

Different encystment media were evaluated for their ability to induce *A. triangularis* WU19001 encystation using the OFAT optimization approach. The encystment media used in this study included NEM medium (pH 8.8) [13], Tris (pH 9.0) [27], and page’s saline (pH 8.0). PYG medium (pH 7.0) was used as the negative control. The encystation assay was performed using 96-well tissue culture plates (SPL Life Science Co., Ltd., Gyeonggi-do, Korea). *A. triangularis* trophozoites grown in PYG medium for 24 h were centrifuged at 1520× *g* for 5 min and washed twice with the test encystment medium to remove residual PYG medium. Trophozoites were adjusted to a final concentration of 3 × 10^5^ trophozoites/mL and transferred into 96-well tissue culture plates containing 150 μL of different encystment media. Encystation was induced by incubation in the dark at 28°C. The encystation efficiency was evaluated by counting the number of cysts at 24, 48, and 72 h after exposure to different encystment media using a hemocytometer (Tiefe Depth Profondeur, Marienfeld, Germany) under an inverted microscope (Nikon ECLIPSE TE2000-S, Tokyo, Japan). The encystation rate was calculated using the following equation:

Encystation rate (%) = (Number of cysts/Total number of cells) × 100

### Optimization of the medium and conditions for inducing *A. triangularis* encystation using a statistical design

To determine the optimal conditions and the best medium for inducing *A. triangularis* encystation, we applied a central composite design (CCD) using three independent variables: glucose, MgCl_2_, and initial pH. These variables were tested at five levels (−1.682, −1, 0, +1, and +1.682), as shown in Table 1. The CCD consisted of 2*^n^* factorial runs with 2*n* axial and *n*_c_ center runs (six replicates) following the methodology described in Chuprom *et al*. [20]. A total of 20 experiments were conducted, including six axial, eight factorial, and six central points. The total number of experiments (N) was calculated using the following equation:


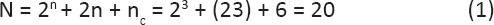


Where *N* denotes the total number of experiments, and *n* denotes the number of factors.

**Table 1:** Coded and actual levels of the independent variables for the design of CCD experiment.

Variables	Units	Symbol coded	Coded variable levels				

			–1.682	–1	0	+1	+1.682
Glucose	% (w/v)	*X*_1_	4.64	6	8	10	11.36
MgCl_2_	mM	*X*_2_	0	2	5	8	10.05
Initial pH		*X*_3_	7.32	8	9	10	10.68

Experimental designs were generated using Stat-Ease software (Design-Expert 6.0.2 Trial, Stat-Ease Corporation, USA). The results obtained from the CCD model were analyzed using multiple regression techniques, and a second-order polynomial equation was used to express the relationship between the predicted response (Y) and the independent variables. The equations are given below:


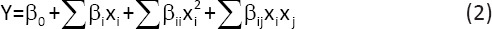


Where Y is the predicted response, β_0_ is the intercept term, β_i_ represents the linear coefficients, β_ii_ represents the quadratic coefficients, β_ij_ represents the interactive coefficients; and x_i_ and j represent the coded independent variables.

### Experimental validation

To validate the response-surface model and confirm the results of the response-surface analysis, we conducted an experimental validation using the optimized conditions.

### CFW staining of mature cysts

*A. triangularis* cysts grown in encystment medium were harvested by centrifugation at 1520× *g* for 5 min and washed twice with encystment medium. *A. triangularis* cysts were then resuspended in 2.5% (v/v) CFW staining solution (Sigma-Aldrich, MA, USA) and incubated in the dark at 28°C for 120 min. After incubation, the cysts were collected again by centrifugation at 1520× *g* for 5 min, washed twice with 0.01 M phosphate-buffered saline (PBS) solution at pH 7.4 to remove excess CFW stain, and resuspended in the same buffer solution. Next, 20 μL of the cyst suspension was placed on a slide, carefully covered with a coverslip, and examined under an Olympus BX-53 fluorescent microscope (Olympus, Tokyo, Japan) with 405-nm excitation and a 420–480-nm emission bandpass.

### Production of dehydrated powder medium for *Acanthamoeba* encystation induction using a spray dryer

An optimized encystment medium formula without the addition of glucose was prepared to produce a dehydrated medium powder for inducing encystation in various *Acanthamoeba* species. The liquid medium was then dried using a Buchi Mini Spray Dryer B-290 (Büchi Labortechnik AG, Switzerland) under the following conditions: an inlet drying air temperature of 195°C, an aspirator rate of 35 m^3^/h, and a feed pump rate of 5.1 mL/min. The yield of the obtained dehydrated medium powder was evaluated.

### Evaluation of the optimized dehydrated medium for inducing *Acanthamoeba* spp. encystation

The dehydrated medium powder was evaluated for its ability to induce encystation in different strains of *Acanthamoeba* spp., as described above. Powder of dehydrated medium with varying glucose concentrations was prepared and autoclaved at 121°C for 15 min. *Acanthamoeba* trophozoites of different strains grown in PYG medium for 24 h were centrifuged at 1520× *g* for 5 min. The trophozoites were adjusted to a final concentration of 3 × 10^5^ trophozoites/mL and transferred into 96-well tissue culture plates containing 150 μL of encystment medium. Encystation was induced by incubating plates in the dark at 28°C. Encystment efficiency was evaluated by counting the number of cysts at 24, 48, and 72 h after exposure to encystment media using a hemocytometer under an inverted microscope.

### Statistical analysis

All experiments were conducted in triplicate, and the data are expressed as mean ± standard deviation (SD). Statistical analysis was performed using one-way analysis of variance (ANOVA) followed by Tukey’s multiple comparison test to evaluate significant differences between groups. A p-value < 0.05 was considered statistically significant.

For medium optimization, a central composite design (CCD) was employed to investigate the effects of three independent variables – glucose concentration, MgCl_2_ concentration, and initial pH – on the encystation rate. The CCD consisted of factorial runs, axial points, and center points, generating a total of 20 experimental runs. The results were analyzed using regression techniques to develop a second-order polynomial equation, describing the relationship between the independent variables and the response (encystation rate). The model’s validity and adequacy were assessed through analysis of variance, with R² and adjusted R² values calculated to ensure model reliability.

Graphical representations of the response surface and contour plots were generated using Design-Expert software (Stat-Ease Inc., Minneapolis, USA) to visualize the effects of variable interactions. The model’s predictive capability was confirmed through experimental validation under optimal conditions. All statistical computations and visualizations were performed using SPSS (version 26.0) and Design-Expert software.

## RESULTS

### Effects of different encystment media on *Acanthamoeba* cyst formation

**Figure 1:**
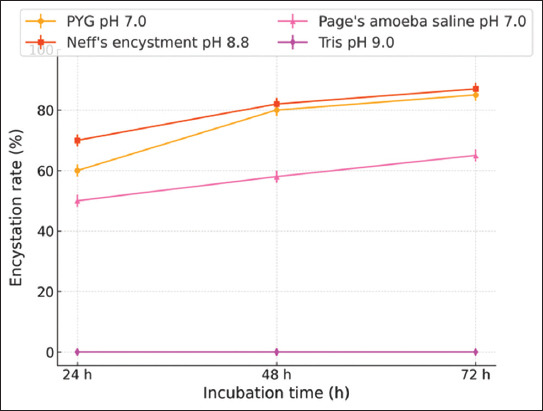
*Acanthamoeba triangularis* WU19001 encystation rates after exposure to various encystment media. The encystation rate was determined after incubating in the dark at 28°C for 24, 48, and 72 h. Values are presented as mean ± SD (n = 3).

**Figure 2:**
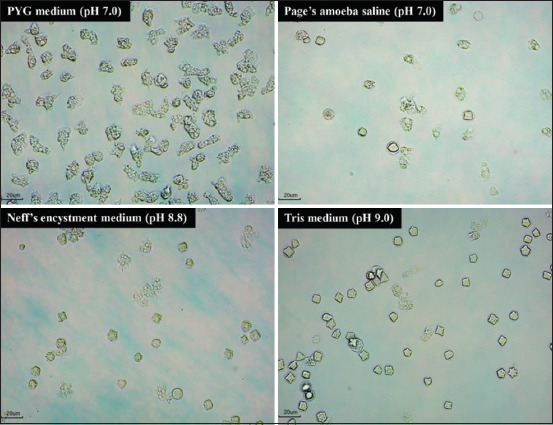
*Acanthamoeba triangularis* WU19001 encystation after exposure to various encystment media observed under an inverted microscope at a magnification of 200×. The incubation was carried out for 72 h in the dark at 28°C.

**Figure 3:**
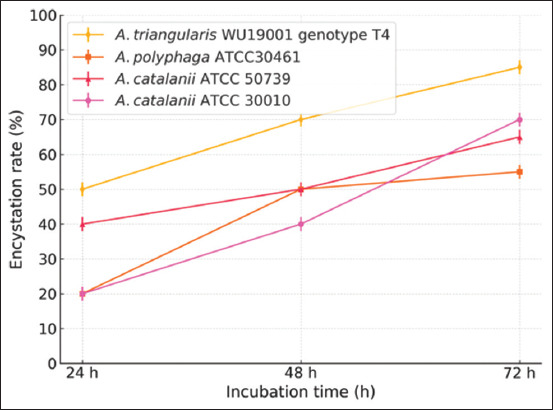
Encystation rates of *Acanthamoeba* spp. after cultivation in Tris medium (pH 9.0). The encystation rates were measured after incubation in the dark at 28°C for 24, 48, and 72 h. Values are presented as mean ± SD (n = 3).

**Figure 4:**
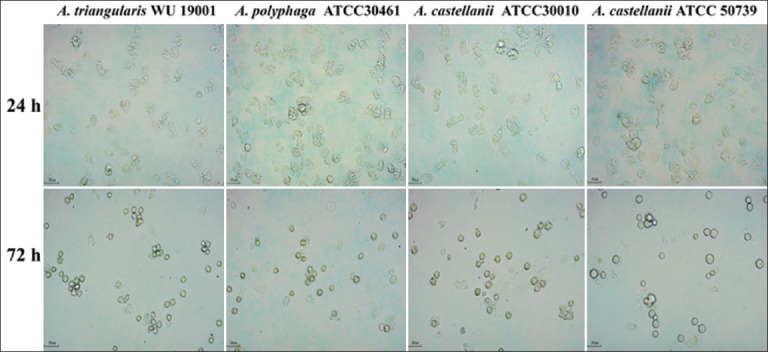
Encystation of *Acanthamoeba* spp. after cultivation in Tris medium (pH 9.0) observed under an inverted microscope (200×) after incubation for 24 and 72 h in the dark at 28°C.

The effectiveness of different encystment media in inducing *A. triangularis* cyst formations was evaluated using a OFAT non-statistical experimental approach. The results demonstrated that NEM (pH 8.8) and Tris medium (pH 9.0) were the most effective encystment media for inducing mature *A. triangularis* cysts. After 72 h of incubation in the dark at 28°C in NEM and Tris medium, *A. triangularis* trophozoites were successfully encysted with encystation rates of 83.87% and 82.80%, respectively (Table 2, Figures 1 and 2). In addition, *A. triangularis* exhibited a relatively low encystation rate of 56.99% in page’s amoeba saline solution (pH 7.0) after 72 h of incubation in the dark at 28°C. Furthermore, the effectiveness of Tris medium (pH 9.0) in inducing the encystation of different strains of *Acanthamoeba* spp. was evaluated. As shown in Figures 3 and 4, the encystation rates of *A. castellanii* ATCC30010, *A. castellanii* ATCC50739, and *A. polyphaga* ATCC30461 cultured in Tris medium were 66.77%, 53.06%, and 57.67%, respectively. Tris medium was chosen as the base medium for developing a novel encystment medium based on its favorable encystation ability and cost considerations.

**Table 2:** The encystation rate of *A. triangularis* WU19001 genotype T4 after exposed to different encystment media in the dark at 28°C for 24–72 h.

Media	Encystation rate (%)		

	24 h	48 h	72 h
PYG pH 7.0	0.00 ± 0.00^Ad^	0.00 ± 0.00^Ac^	0.00 ± 0.00^Ac^
Neff’s encystment pH 8.8	59.14 ± 3.72^Ba^	78.49 ± 4.93^Aa^	83.87 ± 3.23^Aa^
Page’s amoeba saline pH 8.0	41.94 ± 3.23^Bc^	45.16 ± 3.23^Bb^	56.99 ± 1.86^Ab^
Tris pH 9.0	51.61 ± 3.23^Cb^	72.04 ± 1.86^Ba^	82.80 ± 3.72^Aa^

Values are given as mean ± SD (n = 3). Different capital letters (A, B, C) in the same row indicate significant values among each medium for different incubation times, while different small letters (a, b, c, and d) indicate significant values at each time for different media (p < 0.05)

### Rapid encystation of *A. triangularis* cultured in optimized encystment medium

To obtain large numbers of mature and synchronous cysts, a modified Tris medium was developed using RSM to optimize the concentration of each component. A CCD with three independent variables (glucose, MgCl_2_, and initial pH) at five levels was used to create a novel encystment medium for rapid *A. triangularis* encystation (Table 1). Table 3 reveals that experimental run no. 15–20 exhibited the highest encystation rate, ranging from 99% to 100%, with the following conditions: 8% (w/v) glucose, 5.0 mM MgCl_2,_ and an initial pH of 9.0. The Design-Expert® software version 13 (Stat-Ease Inc.) was used to generate a quadratic mathematical model (Eq. 3), which included all terms regardless of their significance level.


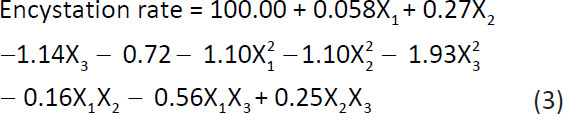


Where X_1_, X_2_, and X_3_ are the coded variables for glucose, MgCl_2_, and the initial pH, respectively.

**Table 3:** Central composite design matrix for the experimental design along with actual and predicted responses for the encystation rate (%) of *Acanthamoeba triangularis* WU19001 genotype T4.

S. No.	*X*_1_ (Glucose, %)	*X*_2_ (MgCl_2_, mM)	*X*_3_ (Initial pH)	Encystation rate (%)	

				Actual	Predicted
1	6	2	8	96.39	96.60
2	10	2	8	98.47	98.15
3	6	8	8	97.20	96.95
4	10	8	8	97.09	97.88
5	6	2	10	96.15	94.94
6	10	2	10	94.41	94.24
7	6	8	10	96.38	96.28
8	10	8	10	95.59	94.96
9	4.64	5	9	97.26	97.86
10	11.36	5	9	98.06	98.06
11	8	0	9	95.76	96.45
12	8	10.05	9	97.44	97.35
13	8	5	7.32	96.91	96.46
14	8	5	10.68	91.56	92.61
15	8	5	9	100.02	100.00
16	8	5	9	99.90	100.00
17	8	5	9	100.10	100.00
18	8	5	9	100.20	100.00
19	8	5	9	99.97	100.00
20	8	5	9	99.90	100.00

The model (following ANOVA analysis) demonstrated a high R^2^ value of 94.82%, indicating that only 5.18% of the variation could not be accounted for by this model (Table 4). The adjusted R^2^ value of 90.16% further confirmed the model’s reliability and agreement with the observed and predicted encystation rates. The coefficient of variation for encystation rate was 0.72%, indicating excellent reproducibility.

**Table 4:** Results of the regression analysis of the second-order polynomial model for optimization of encystation rate.

Source	Sum of squares	*DF*	Mean square	*F*-value	*p*-value prob >*F*
Model	90.73	9	10.08	20.35	< 0.0001
Residual	4.95	10	0.50		
Lack of fit	4.88	5	0.98	69.82	0.0001
Pure error	0.070	5	0.014		
Total correlation	95.68	19			

*R*^2^=0.9482, *R*^2^_adj_=0.9016, Pre *R*^2^=0.5914, Adequate Precision=14.844, C.V. = 0.72

**Figure 5:**
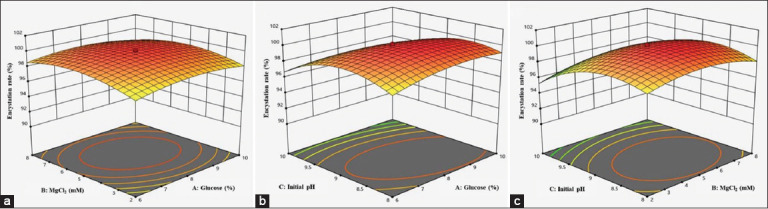
Response surface 3D contour plots of *Acanthamoeba triangularis* WU19001 encystation rate. (a) Glucose and MgCl_2_, (b) Glucose and initial pH, and (c) MgCl_2_ and initial pH were varied while keeping another variable constant (being the 3 independent variables: glucose, MgCl_2_, and initial pH).

Three-dimensional (3D) response surface plots (Figure 5) were used to illustrate the relationships between the response (encystation rate) and experimental levels of each variable. Figure 5a shows that the maximum encystation rate of *A. triangularis* WU19001 was achieved at the mean levels of glucose and MgCl_2_, whereas further increases in these variables resulted in a gradual decrease in yield. Figure 5b shows that the highest encystation rate was observed at pH 9.0 and a glucose concentration of 8% (w/v).

**Figure 6:**
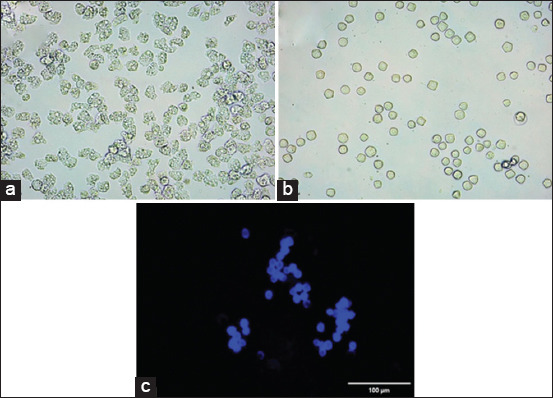
*Acanthamoeba triangularis* WU19001 encystation after exposure to the novel (optimized) encystment medium. (a) Trophozoites of *A. triangularis* WU19001 exposed to PYG medium for 72 h, captured under an inverted microscope (200×). (b) Production of *A. triangularis* cysts after exposure to the novel encystment medium for 72 h, captured under an inverted microscope (200×). (c) Fluorescent image (200×) of an *A. triangularis* cyst after exposure to the novel encystment medium for 72 h.

The suggested optimal conditions (8.31% w/v glucose, 5.23 mM MgCl_2_, and an initial pH of 8.69) were tested in triplicate to validate the optimization results. Under these conditions, the encystation rate was 98.78%, which closely matched the predicted value (Table 5) with a slight error of 2.41% compared with the actual values. This indicates that the predicted optimal conditions were reasonably accurate and reliable, with a slight discrepancy between the predicted and actual encystation rates. However, in the best run (runs no. 15–20, Table 3), the encystation rate ranged from 99% to 100%. Figure 6 depicts the triggering of *Acanthamoeba* trophozoites (Figure 6a) and the resulting mature cysts (Figures-6b and c) under the best-run conditions. CFW staining was used to visualize *Acanthamoeba* cysts. It revealed a double layer of CFW-stained cells in mature cysts (Figure 6c). The encystation rate in the verified set (8.31% w/v glucose, 5.23 mM MgCl_2_, and an initial pH 8.69) was lower than that of the best run (runs no. 15–20) (8.0% w/v glucose, 5.0 mM MgCl_2_, and an initial pH 9.0). Based on these findings, the best run conditions (runs no. 15–20) were selected for further investigation.

**Table 5:** Verification test based on optimal conditions predicted by central composite design for encystation rate.

Condition	Encystation rate (%)		Error (%)

	Actual	Predicted	
Predicted condition	98.78	100.19	2.41
8.31% (w/v) glucose			
5.23 mM MgCl_2_			
pH 8.69			

### Production of dehydrated powder medium

**Figure 7:**
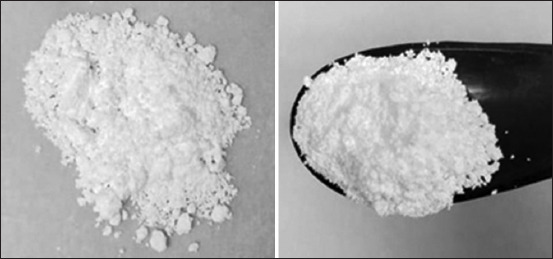
Novel dehydrated encystment powder medium. Powder was obtained using a spray dryer.

Based on CCD results, a novel, optimized encystment medium was developed and prepared. The preparation process involved sterilization by autoclaving at 121°C for 15 min, followed by drying using a spray dryer under the following conditions: Inlet drying air temperature of 195°C, aspirator rate of 35 m^3^/h, and feed pump rate of 5.1 mL/min. The resulting spray-dried medium exhibited a yield of 6 g/L from the liquid medium. The obtained dehydrated medium powder had a white appearance and a moisture content of 7.29% (Figure 7).

### Dehydrated encystment medium powder for inducing *Acanthamoeba* spp. encystation

**Figure 8:**
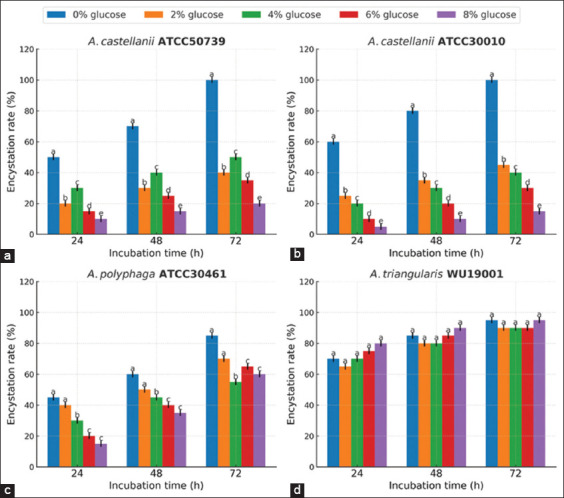
Encystation rates of *Acanthamoeba* spp. after exposure to the novel encystment medium with varying concentrations of glucose. The encystation rate was determined after incubation in the dark at 28°C for 24, 48 and 72 h. Values are presented as mean ± SD (n = 3). Different letters (a, b, c, and d) indicate significantly different values (p < 0.05) among different glucose concentrations.

**Figure 9:**
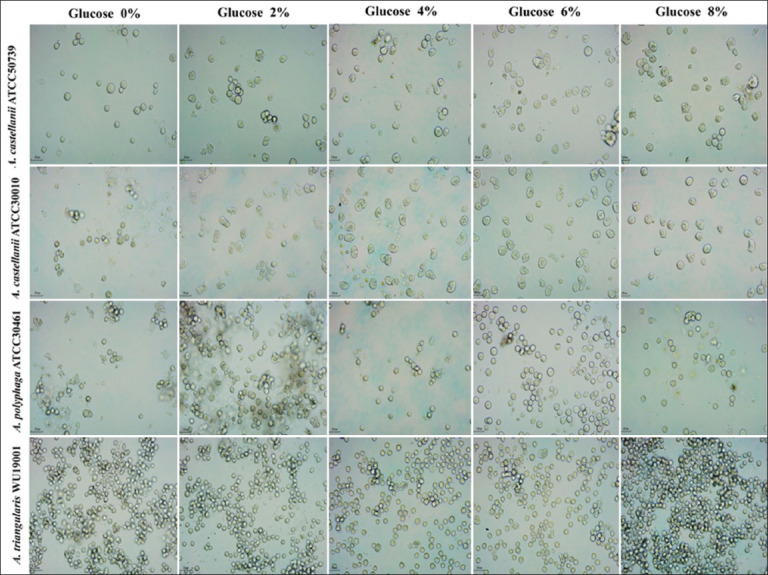
*Acanthamoeba* spp. after exposure to the novel encystment medium with varying concentrations of glucose. The encystation process was observed using an inverted microscope (200×) following 72 h of incubation in the dark at 28°C.

To evaluate the effectiveness of the novel encystment medium in inducing *Acanthamoeba* spp. encystation, 0.6 g of this medium powder was mixed with 100 mL of distilled water, and different concentrations of glucose (0, 2, 4, 6, and 8% w/v) were added and sterilized. After incubation for 72 h, it was found that *A. castellanii* ATCC50739 and *A. castellanii* ATCC30010 showed the highest encystation rates in the encystment medium containing 0% (w/v) glucose, with encystation rates of 98.77% and 90.98%, respectively (Figures 8 and 9). *A. polyphaga* ATCC30461 exhibited the highest encystation rate in the encystment medium containing 6% (w/v) glucose, with an encystation rate of 90.38%. For *A. triangularis* WU19001, a high encystation rate of 98.07% was observed in the encystment medium containing 8% (w/v) glucose (Figures 8 and 9). However, no significant difference (p > 0.05) was found in the encystation rate of *A. triangularis* when exposed to the encystment medium with different glucose concentrations (0%–8% glucose).

## DISCUSSION

Scientific and medical communities have started to pay more attention to *Acanthamoeba* spp. because the organism has shown the ability to cause severe infections in humans and animals [4]. One of the key research areas surrounding *Acanthamoeba* spp. is its encystment. *Acanthamoeba* cysts are a significant concern in the medical field because they can resist most standard treatments, including antibiotic and antifungal treatments [28]. Therefore, the study of *Acanthamoeba* spp. and its encystment process has great potential for improving our understanding of microbial survival strategies and for developing novel treatment strategies for *Acanthamoeba*-related infections.

Various studies have examined *Acanthamoeba* encystment induction using different media [13, 29, 30]. A previous study by Neff *et al*. [13] has identified NEM as the most effective medium for *Acanthamoeba* cyst production. Griffiths and Hughes [29] demonstrated that adding 50 mM MgCl_2_ to a growth medium containing proteose-peptone, yeast extract, and glucose can induce *Acanthamoeba* encystment. While solid media like NNA supplemented with heat-inactivated *Escherichia* *coli* can also be used for *Acanthamoeba* cyst production, this approach has limitations, such as low cyst yields and potential contamination by *E. coli* protein [30].

In this study, different liquid media were evaluated for their efficiency in cyst production using a non-statistical methodology called “OFAT,” with PYG medium serving as a negative control. NEM (pH 8.8) and Tris (pH 9.0) were identified as the most effective encystment media for inducing mature cysts of *A. triangularis* WU19001 in this study. NEM is a well-established medium for cyst production and has been used in many previous studies by De Obeso Fernández del Valle *et al*. [31] and Maal-Bared [32]. However, NEMs often contain expensive components like 2-amino-2-methyl-1,3-propanediol. In addition, Tris medium exhibited a high encystation rate for *A. castellanii* [33]. This study also demonstrated significant encystation rates of *A. castellanii* ATCC30010, *A. polyphaga* ATCC30461, *and A. castellanii* ATCC50739 when exposed to Tris medium. Furthermore, the cost of the Tris medium components was lower than that of the NEMs. Considering these advantages, Tris medium was selected as the base medium for developing a novel encystment medium using RSM with a CCD.

The highest encystation rate was achieved when *A. triangularis* trophozoites were exposed to a modified encystment medium containing 8.0% (w/v) glucose and 5.0 mM MgCl_2_ with an initial pH of 9.0. Glucose and MgCl_2_ were found to have a positive impact on *Acanthamoeba* encystation. Aqeel *et al*. [34] reported that *A. castellanii* encystment was triggered by incubating trophozoites in PBS containing 50 mM MgCl_2_ and 10% (w/v) glucose, which is consistent with our findings. However, the authors suggested that pH 9.0 has an adverse effect on encystation, which is contrary to our results. We also found that encystation can be triggered without altering the medium’s osmolarity. A previous study by Cordingley *et al*. [35] has used NaCl or glucose addition to the growth medium to achieve a specific osmolarity and obtain *Acanthamoeba* cysts. Alkaline pH influences *Acanthamoeba* encystations [18]. In this study, an optimized encystment medium was formulated using Tris-HCl (3.152 g/L), NaCl (5.55 g/L), glucose (8% w/v), and MgCl_2_ (5.0 mM) at an initial pH of 9.0. In contrast, the original Tris medium consisted of Tris-HCl (3.152 g/L), NaCl (5.55 g/L), KCl (0.37 g/L), MgSO_4_ (0.96 g/L), CaCl_2_ (0.044 g/L), and NaHCO_3_ (0.084 g/L). The novel encystment medium exhibited a higher encystation rate than the original medium.

Considering the current unavailability of dehydrated encystment medium powder, which is crucial for achieving the specific osmolarity required for *Acanthamoeba* spp. encystation, we developed a novel dehydrated encystment medium powder without glucose supplementation. This medium was produced using spray drying, resulting in a white powder dehydrated medium.

*A. castellanii* ATCC50739 and *A. castellanii* ATCC30010 demonstrated the highest rates of encystation in the encystment medium without glucose. *A. polyphaga* ATCC30461 exhibited the highest encystation rate with the addition of 6% (w/v) glucose, whereas *A. triangularis* WU19001 demonstrated the highest encystation rate in the medium containing 8% (w/v) glucose. However, no significant differences in encystation rates were observed among the different glucose concentrations. These findings suggest that *Acanthamoeba* spp. encystation occurs at a specific osmolarity [36]. Moreover, increased osmolarity is a significant stimulus for encystation in certain *Acanthamoeba* spp. Cordingley *et al*. [35] reported that osmolarity-induced encystation in *A. castellanii* may involve conformational changes in receptors expressed on the organism’s surface.

## CONCLUSION

This study successfully developed a novel encystment medium for inducing synchronized cyst formation in *Acanthamoeba* spp., addressing the challenges posed by the organism’s dual life stages. The optimized medium, comprising Tris-HCl, NaCl, 8% (w/v) glucose, and 5.0 mM MgCl_2_ at an initial pH of 9.0, demonstrated exceptional efficiency, achieving encystation rates of 99%–100%. In addition, a powdered version of the medium was formulated through spray drying, facilitating ease of storage and transportation. The powdered medium retained its efficacy across various Acanthamoeba strains, with strain-specific glucose concentrations enhancing cyst production. For instance, *A. castellanii* strains exhibited peak encystation in glucose-free conditions, while *A. triangularis* and *A. polyphaga* responded best to 8% and 6% glucose, respectively.

The strength of this study lies in its systematic optimization approach using response surface methodology, which minimized experimental trials while maximizing medium performance. The development of a powdered encystment medium marks a significant advancement, providing a cost-effective and practical tool for laboratory research and diagnostics.

However, the study has certain limitations. While the medium was validated for its efficacy in inducing encystation across several *Acanthamoeba* strains, its performance in field or clinical settings remains untested. In addition, long-term stability studies for the powdered medium under various storage conditions were not conducted.

Future research should focus on evaluating the medium’s applicability in clinical diagnostics, particularly for detecting *Acanthamoeba*-induced infections in human and veterinary samples. Furthermore, exploring the medium’s compatibility with high-throughput screening techniques and its potential for facilitating research on *Acanthamoeba* pathogenesis could yield valuable insights. Long-term stability and scalability studies are also warranted to enhance the commercial viability of this innovation.

## DATA AVAILABILITY

All data generated during the study are included in the manuscript.

## AUTHORS’ CONTRIBUTIONS

VN: Supervised the study. VN and JC: Conceptualized the study and drafted and revised the manuscript. JC, SS, WM, and RB: Produced the figures and tables and performed an experiment of medium optimization. JC, AKP, SMRO, MR, and TOJ: Performed statistical analysis. JC, VN, AKV, MLP, PW, and CW: Interpreted the results and drafted and revised the manuscript. All authors have read and approved the final manuscript.
